# Increased levels of versican and insulin-like growth factor 1 in peritumoral mammary adipose tissue are related to aggressiveness in estrogen receptor-positive breast cancer

**DOI:** 10.1186/s10020-024-00968-8

**Published:** 2024-11-05

**Authors:** Paola Mirra, Alessia Parascandolo, Graziella Marino, Federica D’Alterio, Lorenza Zinna, Antonella Desiderio, Giuseppe Patitucci, Giulia Anna Carmen Vita, Valentina Condelli, Sabino Russi, Francesco D’Andrea, Francesco Beguinot, Claudia Miele, Pietro Formisano, Vittoria D’Esposito

**Affiliations:** 1 URT “Genomic of Diabetes”, Institute for Experimental Endocrinology and Oncology “G. Salvatore”, National Research Council (IEOS-CNR), Via Pansini 5, 80131 Naples, Italy; 2 Department of Translational Medicine, University of Naples “Federico II”, Via Pansini 5, 80131 Naples, Italy; 3 Department of Breast Surgery, Istituto di Ricovero e Cura a Carattere Scientifico (IRCCS) CROB Centro di Riferimento Oncologico della Basilicata, Rionero in Vulture, Italy; 4 Department of Anatomical Pathology, Centro di Riferimento oncologico della Basilicata (IRCCS CROB), Rionero in Vulture, Italy; 5 Laboratory of Preclinical and Translational Research, Istituto di Ricovero e Cura a Carattere Scientifico (IRCCS) CROB Centro di Riferimento Oncologico della Basilicata, Rionero in Vulture, Italy; 6 Department of Public Health, University of Naples “Federico II”, Via Pansini 5, 80131 Naples, Italy

**Keywords:** Mammary adipose tissue, Biomarkers, Breast cancer, Versican, IGF-1, BMI

## Abstract

The adipose tissue (AT) surrounding breast cancer (BC) plays a pivotal role in cancer progression and represents an optimal source for new biomarker discovery. The aim of this work was to investigate whether specific AT factors may have prognostic value in estrogen receptor-positive (ER+) BC. Proteoglycan Versican (VCAN), Insulin-like Growth Factor 1 (IGF1), Reticulon 4B (RTN4), chemokines CCL5 (also known as RANTES) and interleukin 8 (IL-8) are expressed in AT and may play important roles in BC progression. Peritumoral AT and tumoral biopsies were obtained from patients with ER+ BC (N = 23). AT specimens were collected also from healthy women (N = 17; CTRL-AT). The analysis of gene expression by qPCR revealed significantly higher mRNA levels of *VCAN*, *IGF1*, *RTN4*, and *CCL5* in BC-AT compared to the CTRL-AT, and no difference in *IL-8* mRNA levels. *VCAN* positively correlated with patient Body Mass Index (BMI) in BC-AT, while not in CTRL-AT. Moreover, *VCAN* and *IGF1* positively correlated with *RTN4* and negatively with *CCL5*. Interestingly, *VCAN* correlated with tumoral Ki67, while *IGF1* with tumoral *OCT4* that, in turn, correlated with tumoral Ki67 and patient BMI. Thus, peritumoral AT content of *VCAN*, and *IGF1* are related to BC proliferation and aggressiveness.

**Supplementary Information:**

The online version contains supplementary material available at 10.1186/s10020-024-00968-8.

## Background

The tumor microenvironment (TME) in breast cancer (BC) has important functions in tumor behaviour and treatment response, making its pathologic assessment critical for the disease management (Li et al. 2021). In this regard, the latest World Health Organization classification of BC included the histologic evaluation of Tumor-infiltrating lymphocytes, fibrotic foci and lymphovascular invasion in TME as a prognostic parameter. In addition, programmed death-ligand 1 (PD-L1) assessment is now required for the therapy with immune checkpoint inhibitors (Li et al. 2021). Nowadays, several stromal factors have been suggested by preclinical and clinical studies to define BC prognosis and guide the therapeutic approach, by using immunohistochemical (IHC), cellular and molecular methods. Multiple machine-learning algorithms have also been applied to identify TME signature that correlated with survival and immunotherapy response in BC (Li et al. 2022; Zhao et al. 2023). A critical aspect in the study of BC-TME is that it is made mainly by AT which is a plastic tissue, highly influenced by patient metabolic status and by the paracrine effect of surrounding BC (D'Esposito et al. 2020). For instance, we and others have shown that the Insulin-like Growth Factor 1 (IGF1), and the chemokines CCL5 and interleukin-8 (IL-8), which have a documented role in BC progression, are molecules produced by AT and represent key signals in the communication between adipose and BC cells (Li et al. 2021; Ambrosio et al. 2017; D'Esposito et al. 2012, 2016; Ji et al. 2021; Xu et al. 2021). Indeed, stroma-derived IGF1 enhances estrogen receptor-positive (ER+) BC cell growth and induces epithelial IGF-1 expression, leading to a more malignant phenotype (D'Esposito et al. 2012; Christopoulos et al. 2015). The relevance of IGF1 is well known for BC cell malignant phenotype. The IGF1 pathway has also been implicated in BC resistance to hormonal agents, HER targeting, and cytotoxic chemotherapy. Therefore, several clinical trials are currently evaluating the efficacy of IGF1 axis inhibition to overcome these resistance mechanisms (Ianza et al. 2021; Kotsifaki et al. 2024; Maor et al. 2007). Stromal CCL5 is involved in different type of human malignancies. In particular in BC, chemokine CCL5 secreted from Tumor Associated Macrophages contributes to cancer malignancy supporting epithelial–mesenchymal transition and aerobic glycolysis (Lin et al. 2017). Previously, we demonstrated that CCL5 is produced by mammary adipocytes and is involved in triple-negative BC cell invasiveness and dissemination (D'Esposito et al. 2016). IL-8 is well accepted to contribute to tumor survival, invasion, TME angiogenesis, and immune suppression via the activation of several intracellular signaling pathways (Xiong et al. 2022). We provided evidence that IL-8 is produced by both mammary mature adipocytes and adipose mesenchymal stromal/stem cells (MSCs), and contributes to the acquisition of stem features by ER+ BC cells and to the induction of Connective Tissue Growth Factor (CTGF) expression. CTGF expression in tumoral tissue correlates with endocrine resistance in patients with ER+ BC (Ambrosio et al. 2017, 2022).

Thus, IGF1, CCL-5 and IL-8 may be considered as potential biomarkers of BC progression in peritumoral AT.

Literature data have revealed an important role also for the proteoglycan Versican (VCAN), and the Reticulon 4B (RTN4—also known as Neurite Outgrowth Inhibitor B, NOGO-B). VCAN has a relevant function in extracellular matrix (ECM) assembly and in cell signaling through its multiple key protein–protein interactions. VCAN could sustain cancer progression either by having a direct impact on the cancer cell phenotype or by influencing the TME (Wight 2017). Increased VCAN expression levels have been correlated to a more aggressive tumor phenotype and local BC invasiveness. Moreover, VCAN enhanced tumor cell resistance to apoptosis and modulated BC cell resistance to chemotherapeutic agents (Du et al. 2011; Yee et al. 2007). RTN4 is involved in cytoskeleton assembly, cell division, and lipid-metabolism processes in BC cell lines. It promotes tumorigenesis and paclitaxel resistance in in vivo models and its expression in BC samples inversely correlates with patient survival (Pathak et al. 2018). Several studies have demonstrated an important role of RTN4 receptor in BC progression and aggressiveness (Wang et al. 2013; Gao et al. 2018; Jin et al. 2018), however the role of AT-derived RTN4 is largely unknown.

Thus, the breast AT is an endocrine organ that secretes many factors which may control also neoplastic processes. In this work we have focused our attention on peritumoral AT derived *IGF1*, *CCL5*, and *IL-8*, that we previously identified as molecules connecting AT and BC. Moreover, we have analysed peritumoral AT VCAN and RTN4 following a large in silico study mainly based on transcriptomic and proteomic data that have reported their presence in mammary AT secretome and their contribution to cancer phenotypes (Fletcher et al. 2017; Lapeire et al. 2017). Here we have evaluated the expression of *IGF1*, *CCL5*, *IL-8*, *VCAN* and *RTN4* in peritumoral AT of women with BC, to investigate their potential role as markers of cancer progression. Notably, in BC, TME shows peculiar molecular signatures depending on ER, PrGR or HER2 status (Cid et al. 2018; Finak et al. 2008; Merlino et al. 2017). Thus, we focused our attention on ER+/HER2− BC which represents the most commonly diagnosed BC subtype (70% of total BC cases) (https://seer.cancer.gov/statfacts/html/breast-subtypes.html).

This study aims to contribute to the field of personalized medicine for the management of BC that is the most prevalent cancer worldwide, with 2.3 million women with BC diagnosis and 685,000 BC—related deaths in 2020 (https://www.who.int/news-room/fact-sheets/detail/breast-cancer). The identification of molecular markers in AT, together with tumoral markers currently used, may pave the way for the setting up of novel and effective tools for BC diagnosis and prognosis, that are urgently needed.

## Methods

### Participants and samples

This was a case–control study of a total of 40 women. This pilot study (Julious 2005) includes a group of women with a diagnosis of ER+ breast cancer (BC) (N = 23) and a group of healthy women with no history of BC (N = 17). For BC patients inclusion criteria were: female gender, age > 18 years, primary ER+ BC diagnosis, understanding and acceptance of informed consent. Exclusion criteria were: neoadjuvant cancer therapy before surgery, refusal to give informed consent. For healthy woman inclusion criteria were: female gender, age > 18 years, understanding and acceptance of informed consent. Exclusion criteria were: the presence of neoplastic and/or endocrine diseases, previous history of cancer, refusal to give informed consent. Paired biopsies of breast tumor tissue and associated mammary AT were obtained from patients during the surgical excision of the tumor. Mammary AT biopsies were obtained from healthy donors during a surgical mammary reduction. Age and Body Mass Index were collected for all study participants. The expression of Estrogen Receptor (ER), Progesterone Receptor (PrGR), Epidermal Growth Factor 2 (HER2) and of the proliferation marker ki67 in tumoral tissues, evaluated by immunohistochemistry by 2 expert pathologists, as routine medical practice, was also noted for BC patients. Informed consents were collected before the surgical procedure. Circulating levels of the Carcinogenic Embryonic antigen (CEA), the Cancer antigen 15-3 (CA 15-3), the Carbohydrate antigen 19-9 (CA 19-9), and the Cancer antigen 125 (CA 125) were gathered for each patient. Protocol was approved by the ethical committees of the IRCCS CROB (n. 2020/00299 16/06/2020) and of the University of Naples “Federico II” (prot. n. 138/16), and according to The Code of Ethics of the World Medical Association (Declaration of Helsinki).

### RNA isolation and analysis

Total RNA was isolated from biopsies of breast tumor tissue and mammary AT using the AllPrep DNA/RNA/miRNA Universal Kit (Qiagen, Hilden, Germany), according to the manufacturer’s instructions. All RNA samples were quantified using the NanoDrop spectrophotometer (Life Technologies, Carlsbad, CA, USA) and RNA quality was evaluated by the ratio of absorbance at the 260/230 nm and the 260/280 nm. 22 BC-AT samples and all CTRL-AT samples reached these standard quality parameters required to be used. Gene expression was determined as previously described (D'Esposito et al. 2021). Briefly, 1 μg of total RNA was reverse transcribed using the SuperScript III Reverse Transcriptase (Thermo Fisher Scientific, Waltham, MA, USA), according to the manufacturer’s instructions. cDNA (10 ng for reaction) was analyzed using the iTaq Universal SYBR Green Supermix (Bio-Rad Laboratories, Hercules, CA, USA), according to the manufacturer’s instructions. All reactions were performed in triplicate in a CFX Connect Real-Time PCR System (Bio-Rad Laboratories, Hercules, CA, USA). The expression of each selected mRNA was quantified as absolute expression units (AU) and Peptidylprolyl Isomerase A (*PPIA*) was used as the housekeeping gene. The specific primer pairs (300 nM each for reaction) used for amplification were: *CCL5 forward* CAGCACGTGGACCTCGCACA, *CCL5 reverse* GGCAGTGGGCGGGCAATGTA, *IGF1 forward* GCAGAACCTGTTTGGCTCTC, *IGF1 reverse* TATGGTCTTTGCAAGGGAGG, *IL-8 forward* TGAGAGTGATTGAGAGTGGA, *IL-8 reverse* TCAAAAACTTCTCCACAACCC, *VCAN forward* GTCTTTACCGCTGTGACGTC, *VCAN reverse* CTGCGTCACACTGCTCAAAT, *RTN4 forward* CCTGCTCTCTGTGACCATCA, *RTN4 reverse* GCGCCTGAGTTCCTTTATCG, *OCT4 forward* TCAGCCACATCGCCCAGCA, *OCT4 reverse* AGGGAAAGGGACCGAGGAG, *PPIA forward* TACGGGTCCTGGCATCTTGT, *PPIA reverse* GGTGATCTTCTTGCTGGTCT. All primer pairs were purchased from Sigma-Aldrich (St. Louis, MO, USA).

### Statistical analysis

Statistical analyses were performed with GraphPad Prism 8.0 software (GraphPad Software Inc., La Jolla, CA). For comparisons between 2 groups, the non-parametric Mann–Whitney test was used. Correlation plots and correlation matrix were obtained with Pearson or Spearman correlation test. Outliers have been detected and removed according to the ROUT method with Q coefficient 1%. p-value of < 0.05 was considered statistically significant.

## Results and discussion

### Anthropometric and clinical characteristics of study population

The enrolled population was represented by 40 subjects, including 23 ER+ BC and 17 healthy women underwent mammary reduction (CTRL). BC patients and healthy CTRLs displayed no statistically significant differences in age and BMI (Table 1). In tumoral specimens, the median expression of ER, PrGR and Ki67 was 85%, 85%, and 21.25%, respectively, as indicated in Table 1. Among 23 ER+ BC patients, 4 of them displayed an ER+/HER2+ subtype (Table 1). For all patients, CEA, CA15-3, CA19-9 and CA125 circulating levels were within the reference range (Table 1).

**Table 1:** Clinical phenotyping of enrolled populations

Parameters (unit)	BC patients (23)	CTRL subjects (17)	P-value
Age (years)	44.6 ± 4	44.4 ± 11	n.s
BMI (kg/m^2^)	25.5 ± 4.9	28.2 ± 4.7	n.s
ER+ (%)	85% (30;95)	–	–
PrGR (%)	85% (3;95)	–	–
Ki67 (%)	21.25% (12.5; 67.8)	–	–
HER2; yes (%)	4 (17%)	–	–
CEA (ng/ml)	1.7 ± 0.8	–	–
CA 15-3 (U/ml)	12.4 ± 6.8	–	–
CA 19-9 (U/ml)	5.5 ± 3.5	–	–
CA 125 (U/ml)	13.4 ± 6.9	–	–

Age, BMI, CEA, CA 15-3, CA 19-9 and CA 125 are expressed as mean ± SD. Patients with HER2 positivity are indicated as number and percentage. Other data are expressed as median and range (min; max)

### *VCAN*,* IGF1*, *RTN4* and *CCL5* expression in mammary adipose tissue

**Fig. 1:**
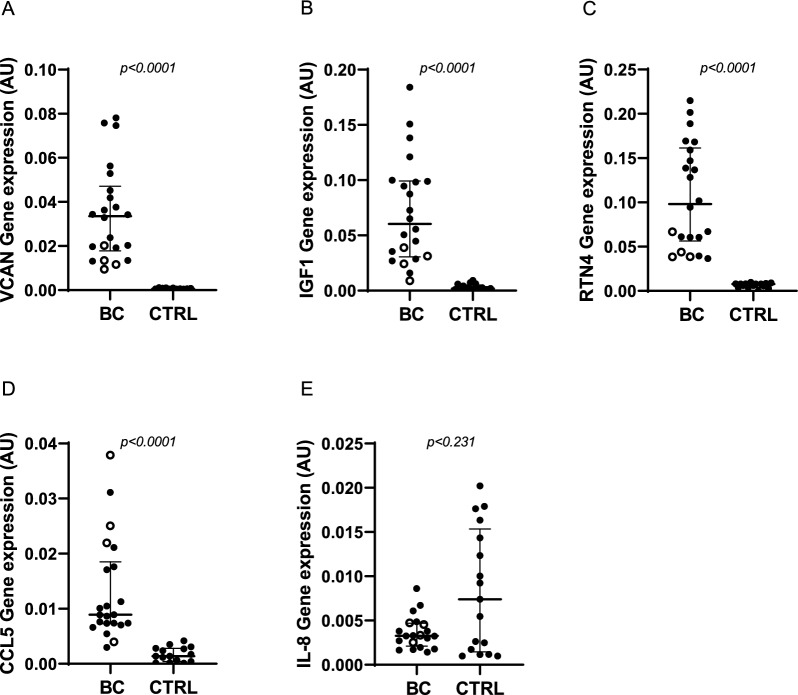
Adipose tissue expression of *VCAN*, *IGF1*, *RTN4*, *CCL5* and *IL-8*. mRNA expression levels of *VCAN* (**A**), *IGF1* (**B**), *RTN4* (**C**), *CCL5* (**D**) and *IL-8* (**E**) were determined in peritumoral adipose tissue of patients with breast cancer (BC-AT) and in mammary adipose tissue of healthy women (CTRL-AT) by qPCR. Data were normalized on the peptidyl-prolyl cis–trans isomerase A (*PPIA*) gene as internal standard. Individual values are expressed in absolute units (AU) and are represented by scatter plots. Full black circles represent ER+/HER2− BC samples while empty circles indicate ER+/HER2+ BC. Scatter plots show median with interquartile range. Data were analyzed using the non-parametric Mann–Whitney test. p values are shown in the graphs

**Fig. 2:**
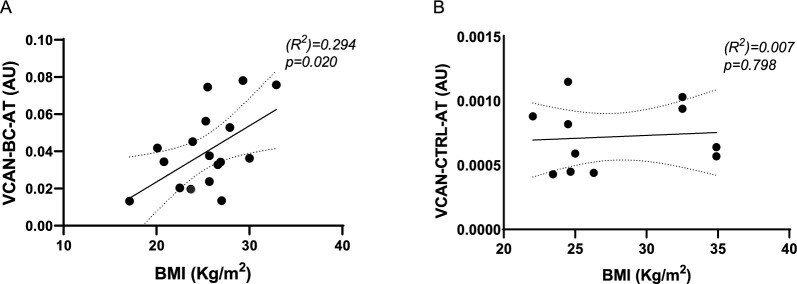
AT–VCAN expression and patient BMI. mRNA expression levels of *VCAN* in BC-AT (**A**) and in CTRL-AT (**B**) were quantified by qPCR. Scatter plots show the association between *VCAN* mRNA levels and the BMI of study participants*.* R^2^ and p values are shown in the graphs. Dashed lines indicate the 95% confidence intervals for the regression line

We evaluated *VCAN*, *IGF1*, *RTN4*, *CCL5* and *IL-8* mRNA levels in both ER+/HER2− and ER+/HER2+ peritumoral AT samples compared to mammary AT from healthy controls. We have provided evidence that *VCAN*, *IGF1*, *RTN4* and *CCL5* displayed a significantly higher expression in BC-AT with respect to CTRL-AT. *IL-8* showed no difference in BC-AT compared to CTRL-AT (Fig. 1). In CTRL-AT *VCAN* mRNA expression levels were detectable in 11 samples, while *CCL5* and *RTN4* in 15 samples. Notably, we observed that ER+/HER2+ samples clustered in the lower region of the scatter plot for VCAN, IGF1 and RTN4 (empty circles, Fig. 1), suggesting that TME is differently influenced by BC subtypes, accordingly to literature evidence (Cid et al. 2018; Finak et al. 2008; Merlino et al. 2017). Thus, we specifically focused our attention on ER+/HER2− subtype. First, we evaluated the correlation between AT genes (*VCAN*, *IGF1*, *RTN4*, *CCL5* and *IL-8*) expression and patient anthropometric and clinical data. As shown in Fig. 2A, we found a positive correlation between *VCAN* and patient BMI in BC-AT (p = 0.020), and no correlation in CTRL-AT (p = 0.798) (Fig. 2B). No significant correlations between other AT genes and donor anthropometric parameters were observed in either BC patients or CTRL subjects (Supplementary Figures 1, 2).

Thus, in the examined population, the analysis of *VCAN* expression in BC-AT has suggested a potential role for this proteoglycan in BC. Murine models revealed that in some cancers VCAN is produced more by the stromal tissue than by cancer cells and contributes to tumor angiogenesis (Asano et al. 2017). VCAN has been detected also in human BC secretome and in multiple in vitro systems (Fletcher et al. 2017). Here, we have shown for the first time that it is highly expressed in BC-AT compared to CTRL-AT in a selected ER+ BC patient population, and that its expression correlates with patient BMI. VCAN plays a pivotal role in inflammatory processes. Obese mice exhibit increased production of VCAN from adipocytes and its inhibition reduces inflammatory gene expression and liver inflammation, resulting in improved insulin sensitivity and glucose tolerance (Han et al. 2020). Recently, serum VCAN levels have been found higher in obese children compared to normal weight control group (Deveci Sevim et al. 2024). VCAN increase in AT of women with cancer and high BMI reflects the ECM remodelling that occurs during obesity and carcinogenesis. The absence of a correlation between *VCAN* and BMI in healthy controls may suggest that tumor could play a role in the modulation of VCAN levels in AT. Thus, VCAN may be envisioned as a molecule with a role in the connection between obesity and BC, even though further studies are still needed.

**Fig. 3:**
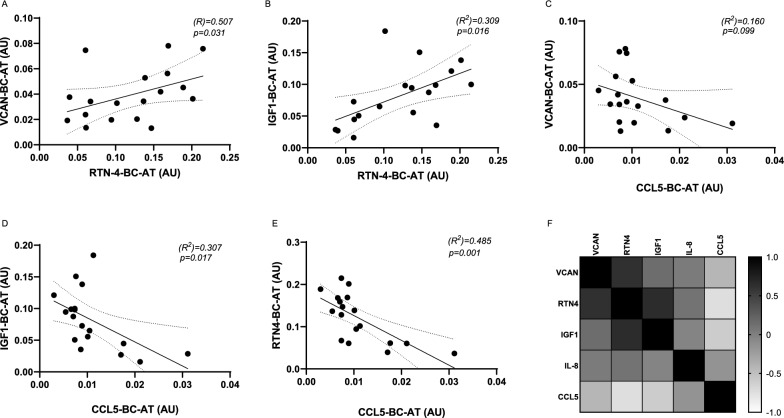
Gene correlation in BC-AT. **A**–**E** mRNA expression levels of *VCAN*, *RTN4*, *IGF1* and *CCL5* in BC-AT were quantified by qPCR. Scatter plots show the association between genes. R^2^ and p values are shown in the graphs. Dashed lines indicate the 95% confidence intervals for the regression line. **F** In the Correlation Matrix, Pearson’s correlation coefficients for gene scaled measurements are visualized by tile-color intensity (according to the legend on the right). Color intensity closer to 1 shows a positive correlation; color intensity closer to − 1 shows a negative correlation; color intensity closer to 0 denotes the absence of a correlation among the considered variables

In BC-AT we found also a positive and significant correlation between *VCAN* and *RTN4* as well as between *IGF1* and *RTN4* mRNA levels, while a negative correlation has been demonstrated for both *VCAN* and *IGF1* with *CCL5* (Fig. 3A–D). Moreover, we observed also a negative and significant correlation between *RTN4* and *CCL5* (Fig. 3E). Thus, in peritumoral AT *VCAN*, *IGF1* and *RTN4* clustered together, while *CCL5* showed an opposite behavior, as also pointed out by the correlation matrix in Fig. 3F.

The correlation of *VCAN* and *IGF1* with the growth factor *RTN4* is a novel finding. Reticulon proteins play a pivotal role in multiple cellular processes. They are involved in the growth and preservation of the Central Nervous System as well as in the development and progression of some neurodegenerative diseases. In addition, through Survivin pathways, *RTN4* promotes cancer cell proliferation. Its role in adipose tissue is largely unknown. However, it has been shown that RTN4 inhibition protects C57BL/6J mice against high-glucose-induced metabolic disorders by ameliorating insulin sensitivity, endoplasmic reticulum stress and inflammation (Pathak et al. 2018; Wang et al. 2013; Kulczynska-Przybik et al. 2021; Zhang et al. 2020a). Thus, its increase in peritumoral AT and its correlation with *VCAN* and *IGF1* may be related to AT rearrangement that occurs and supports BC.

### Peritumoral AT gene correlation with cancer markers

**Fig. 4:**
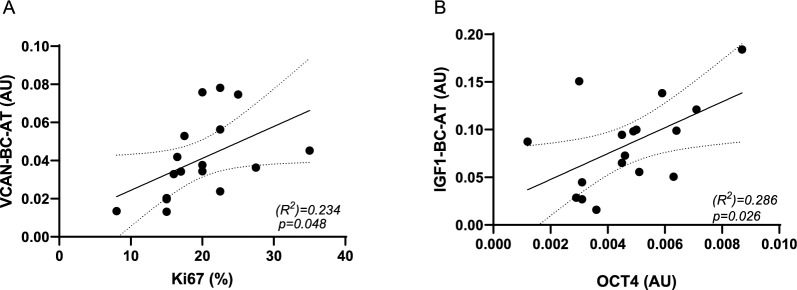
Correlation of BC-AT genes with tumoral markers. mRNA expression levels of *VCAN* and *IGF1* in BC-AT, and of *OCT4* in tumoral tissues were quantified by qPCR. Ki67 levels in tumoral tissues were evaluated by immunohistochemical analysis. Scatter plots show the association between BC-AT *VCAN* mRNA levels with tumoral Ki67 (**A**) and the association between BC-AT *IGF1* with tumoral *OCT4* mRNA levels (**B**). R^2^ and p values are shown in the graphs. Dashed lines indicate the 95% confidence intervals for the regression line

Next, we evaluated the correlation between BC-AT gene mRNA levels and tumoral markers. The expression levels of BC-AT genes did not correlate with the concentrations of circulating tumoral markers (Supplementary Figure 3). However, further studies are needed to investigate this correlation in a cohort of higher-grade BC patients with tumoral marker levels above the reference range. No correlations were also detected for either AT *RTN4* or *IL-8* and tumoral expression of *OCT4*, ER, PrGR and Ki67 (Supplementary Figure 4). Interestingly, peritumoral *VCAN* expression positively correlated with the immunostaining detection of proliferation marker Ki67 in cancer specimens (Fig. 4A). Ki67 is one of the most well-evaluated proliferation markers in tumor cells, with diagnostic and prognostic roles (Sadeghian et al. 2022). Ki67 assessment is routinely used to discriminate the luminal A and luminal B BC subtypes, for the estimation of the prognosis and of the adjuvant treatment choice in ER+ BC (Loibl et al. 2021). However, Ki67 presents some analytical limitations. Therefore, a number of studies are looking for surrogate markers in cancer tissues (i.e. the minichromosome maintenance protein 6, MCM6) (Sadeghian et al. 2022). Future prospective studies are needed to test the possibility of combining Ki67 evaluation in cancer with specific proteins, including VCAN in stroma.

Notably, in this population of ER+ BC patients, Ki67 in tumor inversely correlated with the chemokine *CCL5* (Supplementary Figure 5), which in turn, showed an inverse correlation also with *RTN4*, *IGF1* and *VCAN* in BC-AT. Different studies and dataset analysis have shown a prognostic value for serum and tumoral detection of *CCL5*. However, conflicting results have been reported for molecular detection of CCL5 and its expression seems to be higher especially in advanced disease stages (stages II and III), like in Triple-Negative Breast Cancer (TNBC− negative for ER, PrGR, and HER-2) (Huang et al. 2023; Aldinucci et al. 2020; Brett et al. 2022). Accordingly, we previously demonstrated that CCL5 immuno-detection in peritumoral AT correlated with lymph node and distant metastases, and with reduced overall survival of patients in women with TNBC, whereas no statistically significant associations were found between CCL5 staining and clinical pathological parameters in ER+ cases (D'Esposito et al. 2016). Thus, its involvement in tumor progression may depend on the molecular basis of BC (not always considered) and be strongly related to its cellular source (adipocytes, MSCs, tumoral cells and immune cells) (Aldinucci et al. 2020; Araujo et al. 2018).

**Fig. 5:**
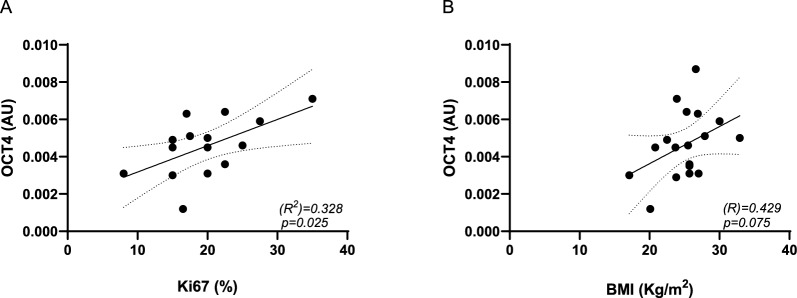
OCT4 correlation with Ki67 and patient + BMI. mRNA expression levels of *OCT4* in tumoral tissues were quantified by qPCR. Scatter plots show the association between *OCT4* mRNA levels with Ki67 (**A**) and patient BMI (**B**). R^2^ and p values are shown in the graphs. Dashed lines indicate the 95% confidence intervals for the regression line

Finally, in this work, we have provided evidence for the first time that *IGF1* mRNA levels in peritumoral AT were positively and significantly correlated to *OCT4* expression detected in paired tumoral biopsies (Fig. 4B) and that in tumoral specimens *OCT4* positively correlated with Ki67 and with BMI of patients (Fig. 5). In BC, OCT4 is the main regulator of cancer stem cells (CSCs), tumor-initiating cells with a pivotal role in cancer recurrence, metastasis, and drug resistance. Noteworthy, patients with higher *OCT4* expression in BC showed poor post-progression survival (Zhang et al. 2020b). Thus, stroma IGF1could be targeted and could represent a marker of BC malignancy.

## Limitations

This is a small size pilot study (Julious 2005). Future studies may use the information arising from this study in their design and extend the analysis to other AT molecules. Retrospective and larger studies are needed to validate these results and to characterize the stroma of other molecular subtypes of BC.

## Conclusion

Peritumoral AT holds several factors potentially useful as biomarkers in BC. In this pilot study on a well-defined population of patients with ER+ cancers we have selected factors of AT derivation with a clear role in BC progression, whose function as TME biomarkers has never been investigated. We have found that VCAN and IGF1 in peritumoral AT correlated with cancer Ki67 and OCT4, respectively, thereby providing an index of cancer proliferation and aggressiveness.

## Supplementary Information

Additional file 1: Supplementary Figure 1. BC-AT gene expression and patient BMI and age. Supplementary Figure 2. CTRL-AT gene expression and patient BMI and age. Supplementary Figure 3. Correlation of BC-AT *VCAN*, *IGF1*, *RTN4*, *CCL5* and *IL-8* with circulating tumoral markers. Supplementary Figure 4. Correlation of BC-AT *RTN4* and *IL-8* with tumoral markers. Supplementary Figure 5. Correlation of BC-AT *CCL5* with tumoral markers.

## Data Availability

The datasets generated and analysed during the current study are available from the corresponding author on reasonable request.

## Abbreviations

AT: Adipose tissue
BC: Breast cancer
BMI: Body Mass Index
CCL5: Chemokine (C-C motif) ligand 5
ECM: Extracellular matrix
ER+: Estrogen receptor-positive
HER-2: Human epidermal growth factor receptor 2
IGF1: Insulin-like growth factor 1
IHC: Immunohistochemical
IL-8: Interleukin-8
MSC: Mesenchymal stromal/stem cell
PrGR: Progesterone receptor
RTN4: Reticulon 4
TME: Tumor microenvironment
TNBC: Triple negative breast cancer
VCAN: Versican proteoglycan

## References

[CR1] Aldinucci D, Borghese C, Casagrande N. The CCL5/CCR5 axis in cancer progression. Cancers. 2020;12(7):1765. 32630699 10.3390/cancers12071765 PMC7407580

[CR2] Ambrosio MR, D’Esposito V, Costa V, Liguoro D, Collina F, Cantile M, et al. Glucose impairs tamoxifen responsiveness modulating connective tissue growth factor in breast cancer cells. Oncotarget. 2017;8(65):109000–17. 29312586 10.18632/oncotarget.22552 PMC5752499

[CR3] Ambrosio MR, Mosca G, Migliaccio T, Liguoro D, Nele G, Schonauer F, et al. Glucose enhances pro-tumorigenic functions of mammary adipose-derived mesenchymal stromal/stem cells on breast cancer cell lines. Cancers. 2022;14(21):5421. 36358839 10.3390/cancers14215421 PMC9655059

[CR4] Araujo JM, Gomez AC, Aguilar A, Salgado R, Balko JM, Bravo L, et al. Effect of CCL5 expression in the recruitment of immune cells in triple negative breast cancer. Sci Rep. 2018;8(1):4899. 29559701 10.1038/s41598-018-23099-7 PMC5861063

[CR5] Asano K, Nelson CM, Nandadasa S, Aramaki-Hattori N, Lindner DJ, Alban T, et al. Stromal versican regulates tumor growth by promoting angiogenesis. Sci Rep. 2017;7(1):17225. 29222454 10.1038/s41598-017-17613-6 PMC5722896

[CR6] Brett E, Duscher D, Pagani A, Daigeler A, Kolbenschlag J, Hahn M. Naming the barriers between anti-CCR5 therapy, breast cancer and its microenvironment. Int J Mol Sci. 2022;23(22):14159. 36430633 10.3390/ijms232214159 PMC9694078

[CR7] Christopoulos PF, Msaouel P, Koutsilieris M. The role of the insulin-like growth factor-1 system in breast cancer. Mol Cancer. 2015;14:43. 25743390 10.1186/s12943-015-0291-7 PMC4335664

[CR8] Cid S, Eiro N, Fernandez B, Sanchez R, Andicoechea A, Fernandez-Muniz PI, et al. Prognostic influence of tumor stroma on breast cancer subtypes. Clin Breast Cancer. 2018;18(1):e123–33. 28927692 10.1016/j.clbc.2017.08.008

[CR9] D’Esposito V, Passaretti F, Hammarstedt A, Liguoro D, Terracciano D, Molea G, et al. Adipocyte-released insulin-like growth factor-1 is regulated by glucose and fatty acids and controls breast cancer cell growth in vitro. Diabetologia. 2012;55(10):2811–22. 22798065 10.1007/s00125-012-2629-7 PMC3433668

[CR10] D’Esposito V, Liguoro D, Ambrosio MR, Collina F, Cantile M, Spinelli R, et al. Adipose microenvironment promotes triple negative breast cancer cell invasiveness and dissemination by producing CCL5. Oncotarget. 2016;7(17):24495–509. 27027351 10.18632/oncotarget.8336 PMC5029717

[CR11] D’Esposito V, Ambrosio MR, Giuliano M, Cabaro S, Miele C, Beguinot F, et al. Mammary adipose tissue control of breast cancer progression: impact of obesity and diabetes. Front Oncol. 2020;10:1554. 32850459 10.3389/fonc.2020.01554 PMC7426457

[CR12] D’Esposito V, Ambrosio MR, Liguoro D, Perruolo G, Lecce M, Cabaro S, et al. In severe obesity, subcutaneous adipose tissue cell-derived cytokines are early markers of impaired glucose tolerance and are modulated by quercetin. Int J Obes. 2021;45(8):1811–20. 10.1038/s41366-021-00850-1 33993191

[CR13] Deveci Sevim R, Gok M, Cevik O, Erdogan O, Gunes S, Unuvar T, et al. Associations of adipocyte-derived versican and macrophage-derived biglycan with body adipose tissue and hepatosteatosis in obese children. J Clin Res Pediatr Endocrinol. 2024;16(2):151–9. 38238969 10.4274/jcrpe.galenos.2024.2023-9-18 PMC11590723

[CR14] Du WW, Yang BB, Yang BL, Deng Z, Fang L, Shan SW, et al. Versican G3 domain modulates breast cancer cell apoptosis: a mechanism for breast cancer cell response to chemotherapy and EGFR therapy. PLoS ONE. 2011;6(11): e26396. 22096483 10.1371/journal.pone.0026396 PMC3212514

[CR15] Finak G, Bertos N, Pepin F, Sadekova S, Souleimanova M, Zhao H, et al. Stromal gene expression predicts clinical outcome in breast cancer. Nat Med. 2008;14(5):518–27. 18438415 10.1038/nm1764

[CR16] Fletcher SJ, Sacca PA, Pistone-Creydt M, Colo FA, Serra MF, Santino FE, et al. Human breast adipose tissue: characterization of factors that change during tumor progression in human breast cancer. J Exp Clin Cancer Res. 2017;36(1):26. 28173833 10.1186/s13046-017-0494-4 PMC5297209

[CR17] Gao P, Wang X, Jin Y, Hu W, Duan Y, Shi A, et al. Nogo-B receptor increases the resistance to tamoxifen in estrogen receptor-positive breast cancer cells. Breast Cancer Res. 2018;20(1):112. 30208932 10.1186/s13058-018-1028-5 PMC6134690

[CR18] Han CY, Kang I, Harten IA, Gebe JA, Chan CK, Omer M, et al. Adipocyte-derived versican and macrophage-derived biglycan control adipose tissue inflammation in obesity. Cell Rep. 2020;31(13): 107818. 32610121 10.1016/j.celrep.2020.107818 PMC7384517

[CR19] Huang Y, Wu L, Sun Y, Li J, Mao N, Yang Y, et al. CCL5 might be a prognostic biomarker and associated with immuno-therapeutic efficacy in cancers: a pan-cancer analysis. Heliyon. 2023;9(7): e18215. 37519664 10.1016/j.heliyon.2023.e18215 PMC10375802

[CR20] Ianza A, Sirico M, Bernocchi O, Generali D. Role of the IGF-1 axis in overcoming resistance in breast cancer. Front Cell Dev Biol. 2021;9: 641449. 33829018 10.3389/fcell.2021.641449 PMC8019779

[CR21] Ji F, Yuan JM, Gao HF, Xu AQ, Yang Z, Yang CQ, et al. Tumor microenvironment characterization in breast cancer identifies prognostic and neoadjuvant chemotherapy relevant signatures. Front Mol Biosci. 2021;8: 759495. 34708079 10.3389/fmolb.2021.759495 PMC8544945

[CR22] Jin Y, Hu W, Liu T, Rana U, Aguilera-Barrantes I, Kong A, et al. Nogo-B receptor increases the resistance of estrogen receptor positive breast cancer to paclitaxel. Cancer Lett. 2018;419:233–44. 29373839 10.1016/j.canlet.2018.01.054 PMC5821135

[CR23] Julious SA. Sample size of 12 per group rue of thumb for a pilot study. Pharm Stat. 2005;4:287–91.

[CR24] Kotsifaki A, Maroulaki S, Karalexis E, Stathaki M, Armakolas A. Decoding the role of insulin-like growth factor 1 and its isoforms in breast cancer. Int J Mol Sci. 2024;25(17):9302. 39273251 10.3390/ijms25179302 PMC11394947

[CR25] Kulczynska-Przybik A, Mroczko P, Dulewicz M, Mroczko B. The implication of reticulons (RTNs) in neurodegenerative diseases: from molecular mechanisms to potential diagnostic and therapeutic approaches. Int J Mol Sci. 2021;22(9):4630. 33924890 10.3390/ijms22094630 PMC8125174

[CR26] Lapeire L, Hendrix A, Lecoutere E, Van Bockstal M, Vandesompele J, Maynard D, et al. Secretome analysis of breast cancer-associated adipose tissue to identify paracrine regulators of breast cancer growth. Oncotarget. 2017;8(29):47239–49. 28525384 10.18632/oncotarget.17592 PMC5564561

[CR27] Li JJ, Tsang JY, Tse GM. Tumor microenvironment in breast cancer-updates on therapeutic implications and pathologic assessment. Cancers. 2021;13(16):4233. 34439387 10.3390/cancers13164233 PMC8394502

[CR28] Li J, Qiu J, Han J, Li X, Jiang Y. Tumor microenvironment characterization in breast cancer identifies prognostic pathway signatures. Genes. 2022;13(11):1976. 36360212 10.3390/genes13111976 PMC9690299

[CR29] Lin S, Sun L, Lyu X, Ai X, Du D, Su N, et al. Lactate-activated macrophages induced aerobic glycolysis and epithelial–mesenchymal transition in breast cancer by regulation of CCL5–CCR5 axis: a positive metabolic feedback loop. Oncotarget. 2017;8(66):110426–43. 29299159 10.18632/oncotarget.22786 PMC5746394

[CR30] Loibl S, Poortmans P, Morrow M, Denkert C, Curigliano G. Breast cancer. Lancet. 2021;397(10286):1750–69. 33812473 10.1016/S0140-6736(20)32381-3

[CR31] Maor S, Yosepovich A, Papa MZ, Yarden RI, Mayer D, Friedman E, et al. Elevated insulin-like growth factor-I receptor (IGF-IR) levels in primary breast tumors associated with BRCA1 mutations. Cancer Lett. 2007;257(2):236–43. 17766039 10.1016/j.canlet.2007.07.019

[CR32] Merlino G, Miodini P, Callari M, D’Aiuto F, Cappelletti V, Daidone MG. Prognostic and functional role of subtype-specific tumor-stroma interaction in breast cancer. Mol Oncol. 2017;11(10):1399–412. 28672102 10.1002/1878-0261.12107 PMC5623822

[CR33] Pathak GP, Shah R, Kennedy BE, Murphy JP, Clements D, Konda P, et al. RTN4 knockdown dysregulates the AKT pathway, destabilizes the cytoskeleton, and enhances paclitaxel-induced cytotoxicity in cancers. Mol Ther. 2018;26(8):2019–33. 30078441 10.1016/j.ymthe.2018.05.026 PMC6094397

[CR34] Sadeghian D, Saffar H, Mahdavi Sharif P, Soleimani V, Jahanbin B. MCM6 versus Ki-67 in diagnosis of luminal molecular subtypes of breast cancers. Diagn Pathol. 2022;17(1):24. 35125121 10.1186/s13000-022-01209-4 PMC8818166

[CR35] Wang B, Zhao B, North P, Kong A, Huang J, Miao QR. Expression of NgBR is highly associated with estrogen receptor alpha and survivin in breast cancer. PLoS ONE. 2013;8(11): e78083. 24223763 10.1371/journal.pone.0078083 PMC3817177

[CR36] Wight TN. Provisional matrix: a role for versican and hyaluronan. Matrix Biol. 2017;60–61:38–56. 27932299 10.1016/j.matbio.2016.12.001 PMC5438907

[CR37] Xiong X, Liao X, Qiu S, Xu H, Zhang S, Wang S, et al. CXCL8 in tumor biology and its implications for clinical translation. Front Mol Biosci. 2022;9: 723846. 35372515 10.3389/fmolb.2022.723846 PMC8965068

[CR38] Xu K, Rahmatpanah F, Jia Z. Editorial: Therapeutic opportunities and innovative biomarkers in tumor microenvironment. Front Oncol. 2021;11: 803414. 34917516 10.3389/fonc.2021.803414 PMC8669590

[CR39] Yee AJ, Akens M, Yang BL, Finkelstein J, Zheng PS, Deng Z, et al. The effect of versican G3 domain on local breast cancer invasiveness and bony metastasis. Breast Cancer Res. 2007;9(4):R47. 17662123 10.1186/bcr1751 PMC2206723

[CR40] Zhang S, Guo F, Yu M, Yang X, Yao Z, Li Q, et al. Reduced Nogo expression inhibits diet-induced metabolic disorders by regulating ChREBP and insulin activity. J Hepatol. 2020a;73(6):1482–95. 32738448 10.1016/j.jhep.2020.07.034

[CR41] Zhang Q, Han Z, Zhu Y, Chen J, Li W. The role and specific mechanism of OCT4 in cancer stem cells: a review. Int J Stem Cells. 2020b;13(3):312–25. 32840233 10.15283/ijsc20097 PMC7691851

[CR42] Zhao H, Yin X, Wang L, Liu K, Liu W, Bo L, et al. Identifying tumour microenvironment-related signature that correlates with prognosis and immunotherapy response in breast cancer. Sci Data. 2023;10(1):119. 36869083 10.1038/s41597-023-02032-2 PMC9984471

