# Supplementary material for: Increased levels of versican and insulin-like growth factor 1 in peritumoral mammary adipose tissue are related to aggressiveness in estrogen receptor-positive breast cancer
Source: Mol Med. 2024 Nov 5;30:201. doi: 10.1186/s10020-024-00968-8 (PMC11539550; doi:10.1186/s10020-024-00968-8)
Supplement: Supplementary file 1 — Additional file 1: Supplementary Figure 1. BC-AT gene expression and patient BMI and age. Supplementary Figure 2. CTRL-AT gene expression and patient BMI and age. Supplementary Figure 3. Correlation of BC-AT VCAN, IGF1, RTN4, CCL5 and IL-8 with circulating tumoral markers. Supplementary Figure 4. Correlation of BC-AT RTN4 and IL-8 with tumoral markers. Supplementary Figure 5. Correlation of BC-AT CCL5 with tumoral markers. [file 10020_2024_968_MOESM1_ESM.pptx]

## Slide 1
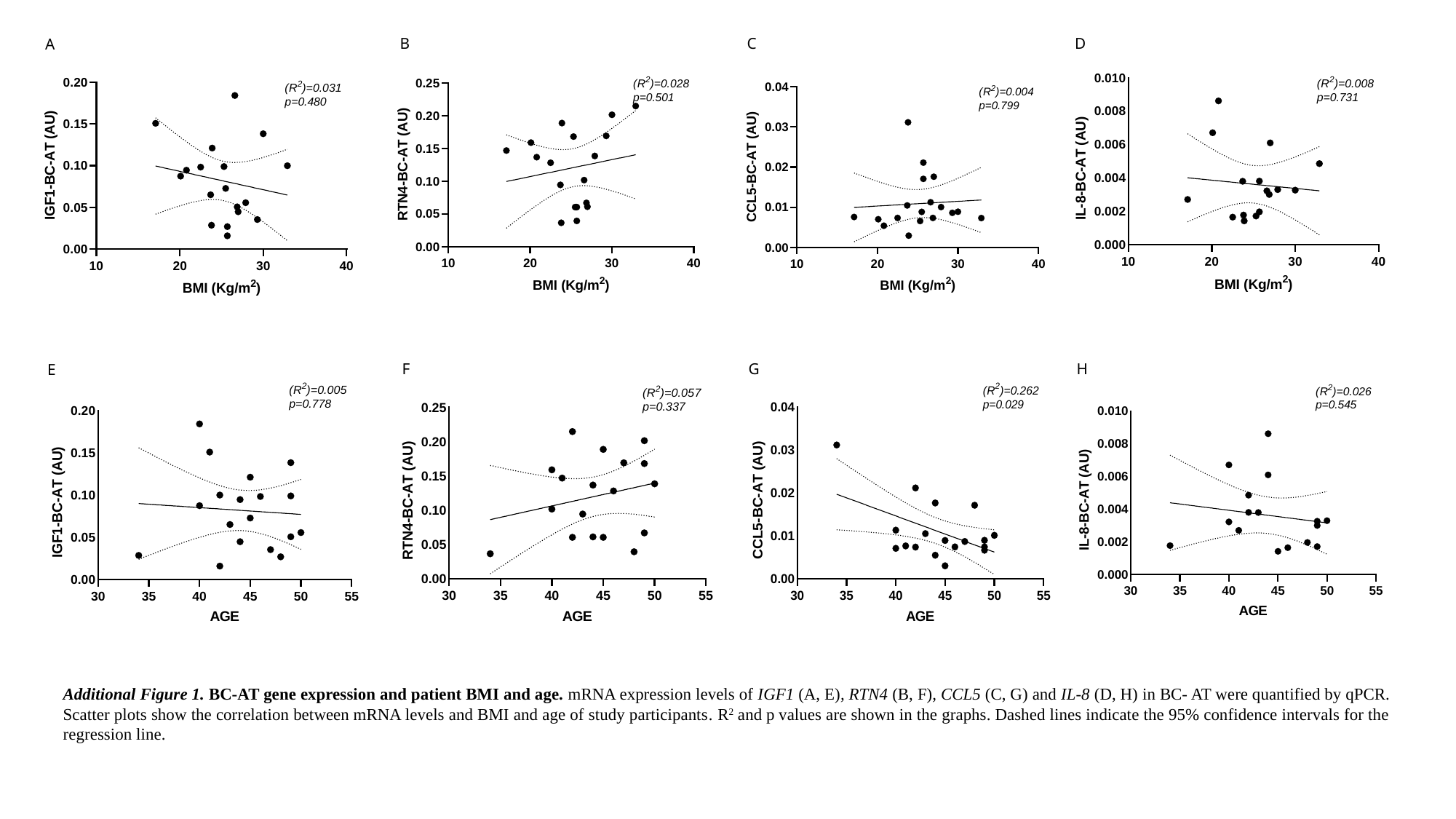

D
C
B
A
H
G
F
E
Additional Figure 1. BC-AT gene expression and patient BMI and age. mRNA expression levels of IGF1 (A, E), RTN4 (B, F), CCL5 (C, G) and IL-8 (D, H) in BC- AT were quantified by qPCR. Scatter plots show the correlation between mRNA levels and BMI and age of study participants. R2 and p values are shown in the graphs. Dashed lines indicate the 95% confidence intervals for the regression line.

## Slide 2
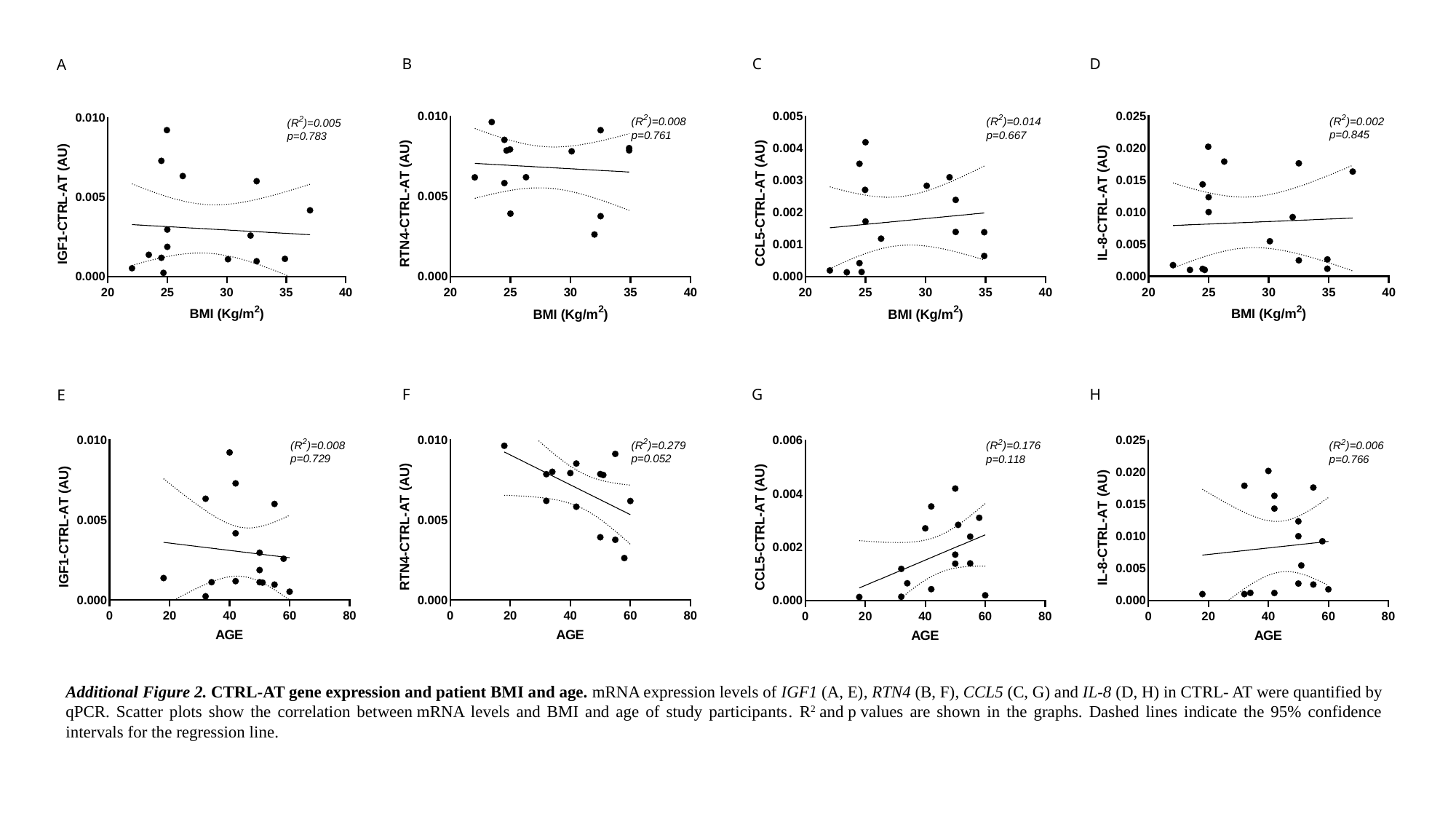

D
C
B
A
H
G
F
E
Additional Figure 2. CTRL-AT gene expression and patient BMI and age. mRNA expression levels of IGF1 (A, E), RTN4 (B, F), CCL5 (C, G) and IL-8 (D, H) in CTRL- AT were quantified by qPCR. Scatter plots show the correlation between mRNA levels and BMI and age of study participants. R2 and p values are shown in the graphs. Dashed lines indicate the 95% confidence intervals for the regression line.

## Slide 3
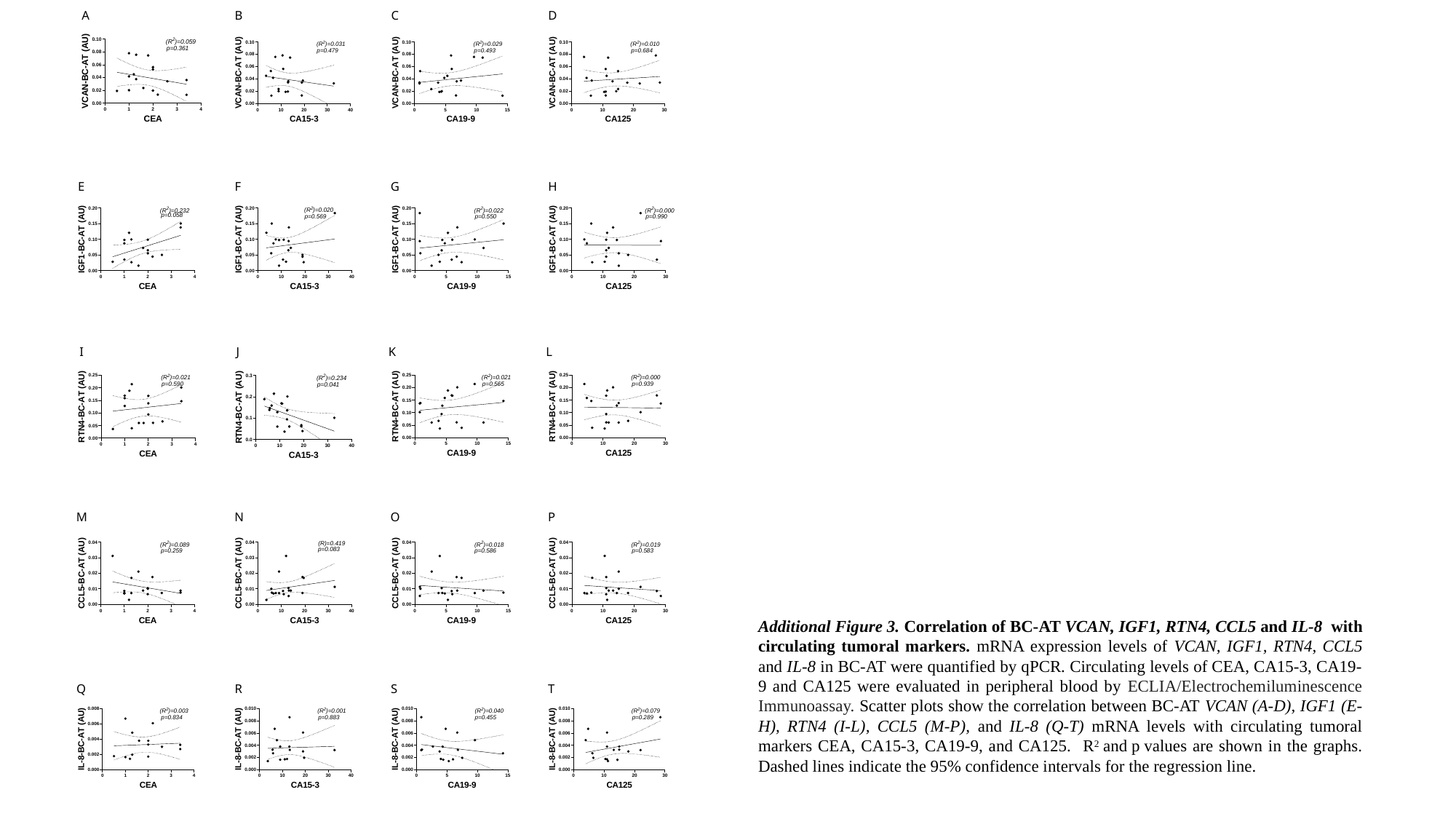

A
B
C
D
E
F
G
H
I
J
K
L
M
N
O
P
Q
R
S
T
Additional Figure 3. Correlation of BC-AT VCAN, IGF1, RTN4, CCL5 and IL-8 with circulating tumoral markers. mRNA expression levels of VCAN, IGF1, RTN4, CCL5 and IL-8 in BC-AT were quantified by qPCR. Circulating levels of CEA, CA15-3, CA19-9 and CA125 were evaluated in peripheral blood by ECLIA/Electrochemiluminescence Immunoassay. Scatter plots show the correlation between BC-AT VCAN (A-D), IGF1 (E-H), RTN4 (I-L), CCL5 (M-P), and IL-8 (Q-T) mRNA levels with circulating tumoral markers CEA, CA15-3, CA19-9, and CA125. R2 and p values are shown in the graphs. Dashed lines indicate the 95% confidence intervals for the regression line.

## Slide 4
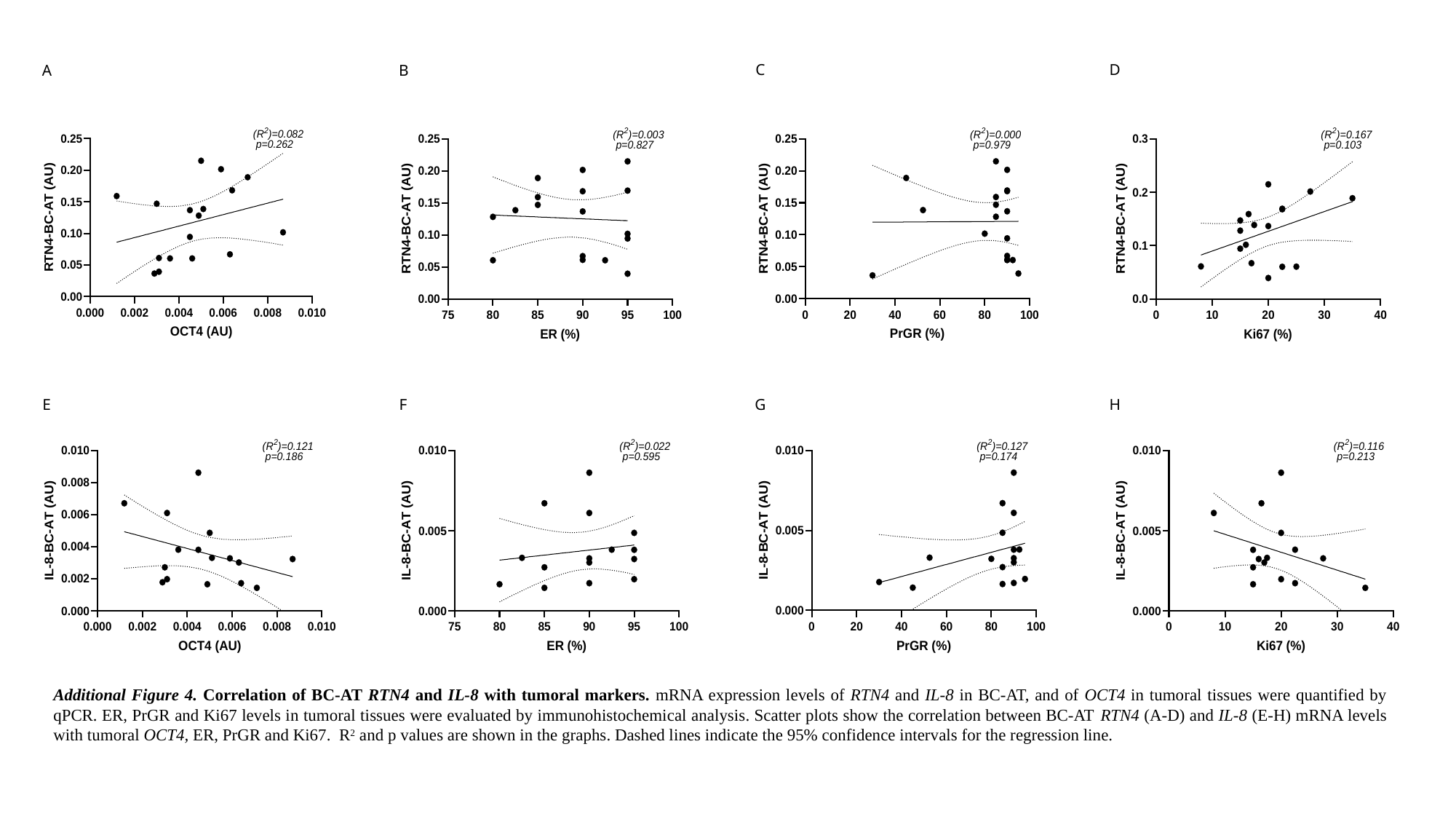

D
C
B
A
H
G
F
E
Additional Figure 4. Correlation of BC-AT RTN4 and IL-8 with tumoral markers. mRNA expression levels of RTN4 and IL-8 in BC-AT, and of OCT4 in tumoral tissues were quantified by qPCR. ER, PrGR and Ki67 levels in tumoral tissues were evaluated by immunohistochemical analysis. Scatter plots show the correlation between BC-AT RTN4 (A-D) and IL-8 (E-H) mRNA levels with tumoral OCT4, ER, PrGR and Ki67. R2 and p values are shown in the graphs. Dashed lines indicate the 95% confidence intervals for the regression line.

## Slide 5
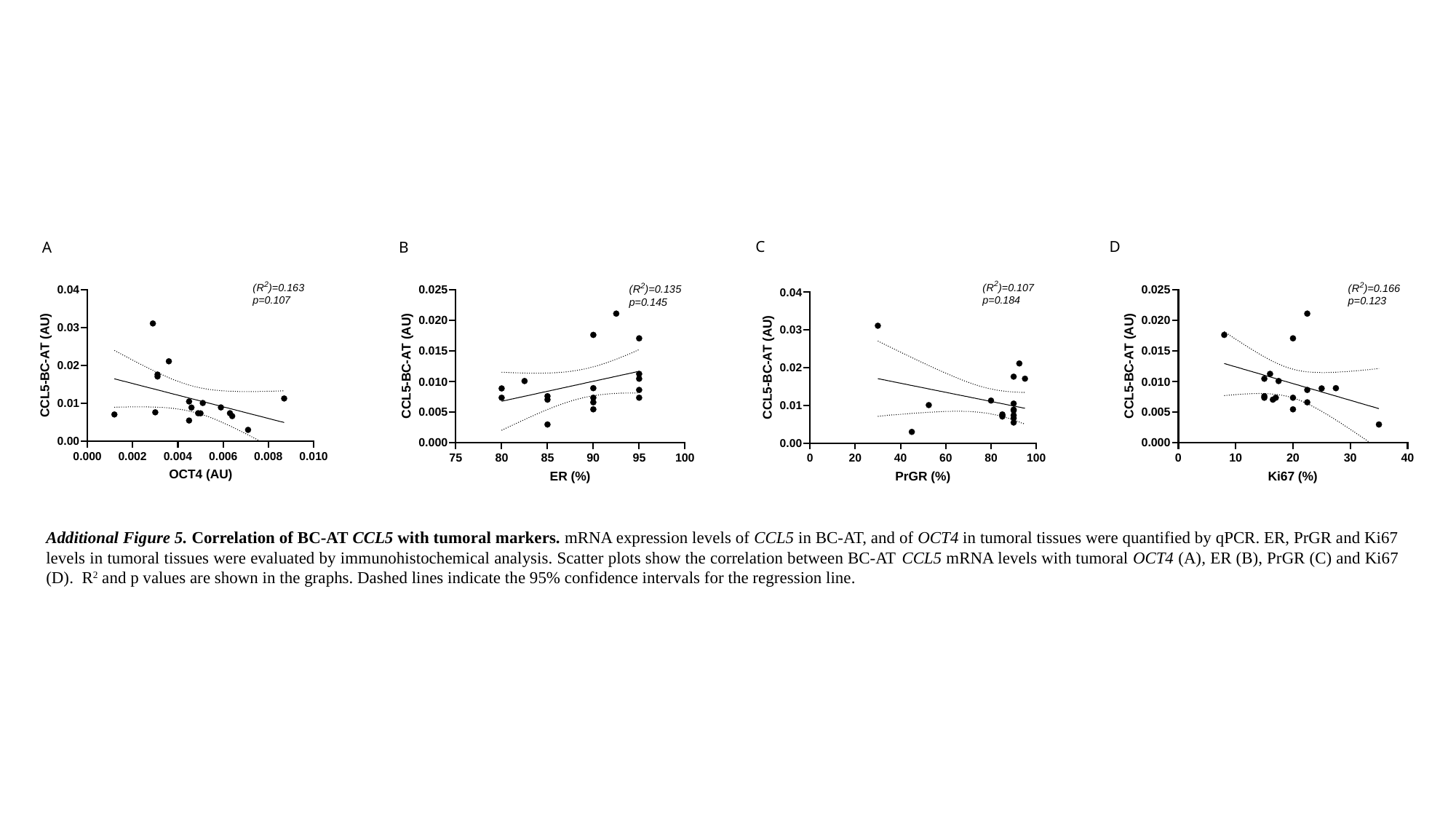

D
C
B
A
Additional Figure 5. Correlation of BC-AT CCL5 with tumoral markers. mRNA expression levels of CCL5 in BC-AT, and of OCT4 in tumoral tissues were quantified by qPCR. ER, PrGR and Ki67 levels in tumoral tissues were evaluated by immunohistochemical analysis. Scatter plots show the correlation between BC-AT CCL5 mRNA levels with tumoral OCT4 (A), ER (B), PrGR (C) and Ki67 (D). R2 and p values are shown in the graphs. Dashed lines indicate the 95% confidence intervals for the regression line.
